# Pulmonary MALT lymphoma: A case report and review of the literature

**DOI:** 10.3892/etm.2014.2072

**Published:** 2014-11-14

**Authors:** LINTAO BI, JUN LI, WANG DAN, ZHENXIA LU

**Affiliations:** Department of Hematology and Oncology, China-Japan Union Hospital Affiliated to Jilin University, Changchun, Jilin 130033, P.R. China

**Keywords:** mucosa-associated lymphoid tissue, lymphoma, pulmonary

## Abstract

Mucosa-associated lymphoid tissue (MALT) lymphoma is an extranodal low-grade B-cell lymphoma. Pulmonary MALT lymphoma is considered to originate from bronchial MALT and is also referred to as bronchial-associated lymphoid tissue lymphoma. Pulmonary MALT lymphoma is a rare disease, but it is the most frequent subset of primary pulmonary lymphoma. The median age at diagnosis of pulmonary MALT lymphoma is 50–60 years, with only few patients aged <30 years. This is the case report of a 19-year-old patient with pulmonary MALT lymphoma presenting with a multiple pulmonary consolidation pattern on computed tomography scans, who underwent successful chemotherapeutic treatment with a chlorambucil-based regimen.

## Introduction

Mucosa-associated lymphoid tissue (MALT) lymphomas were first described in 1983 by Isaacson and Wright, have since been recognized as a separate entity and account for 8–10% of all non-Hodgkin lymphomas (1). Pulmonary MALT lymphoma (pMALToma), also referred to as bronchial-associated lymphoid tissue lymphoma (2), is a rare disease. The development of some pMALTomas has been reported to be associated with chronic inflammation due to autoimmune or infectious diseases (3–4). pMALToma usually has an indolent course and remains localized in the lung for long periods prior to dissemination. The majority of patients with pMALToma have a favorable prognosis. However, the optimal therapy for this rare disease remains under debate and some cases of pMALToma are managed with the watch-and-wait approach. This is the case report of a 19-year-old patient with pMALToma presenting with multiple pulmonary consolidations on computed tomography (CT) scans. The patient received chemotherapy with a chlorambucil-based regimen and achieved a complete remission.

## Case report

**Figure 1:**
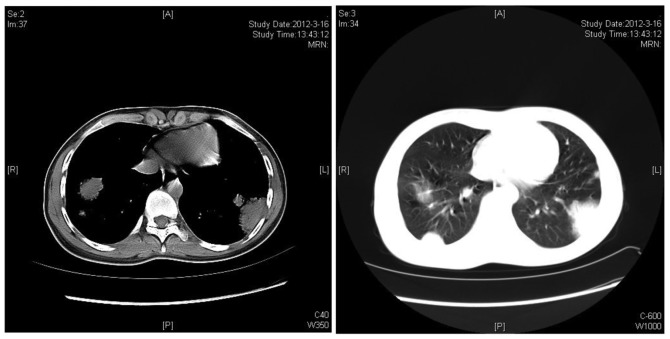
Bilateral pulmonary consolidation on computed tomography.

**Figure 2:**
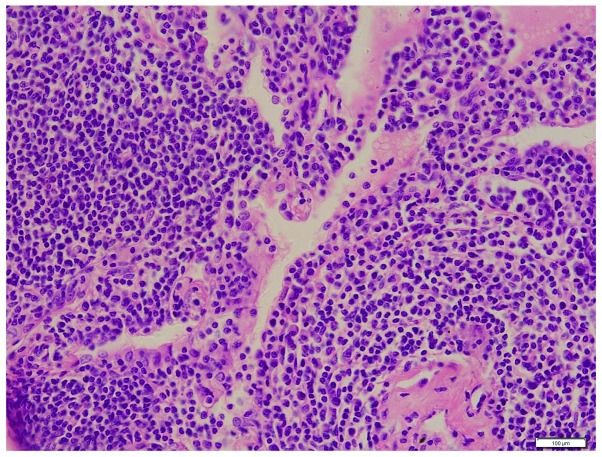
Diffuse infiltration of the lung parenchyma by small lymphocytes, monocytoid cells and plasma cells (magnification, ×20).

**Figure 3:**
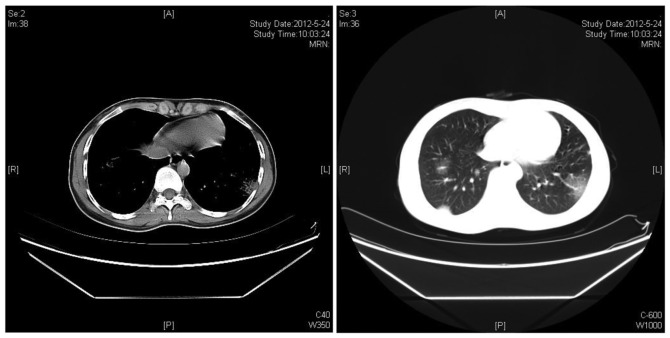
Computed tomography findings following 2 cycles of chemotherapy.

**Figure 4:**
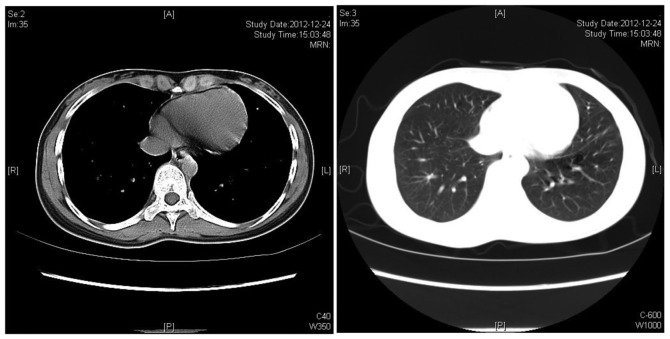
Computed tomography findings following 6 cycles of chemotherapy.

A previously healthy 19-year-old female of Asian ethnic origin, visited our hospital for an evaluation of abnormal chest CT findings on routine physical examination. On admission the patient was asymptomatic, without fever, cough, lymphadenopathy, night sweats or weight loss. As shown in Table I, the patient had normal complete blood count. Additional tests revealed minimally elevated erythrocyte sedimentation rate. The antinuclear antibody test was negative. The levels of lactate dehydrogenase, β_2_ microglobulin and liver function tests were normal. The tests for tuberculosis, human immunodeficiency virus and other viruses, including hepatitis A, B and C, were all negative. The spleen and liver were of normal size and echogenicity. Spiral lung CT revealed bilateral multiple pulmonary consolidations, with the largest lesion sized ~4.5×4.5 cm (Fig. 1). Bone marrow aspiration was negative for lymphoma involvement. The patient underwent CT image-guided biopsy and the diagnosis of pMALToma was confirmed. The microscopic examination revealed lymphoepithelial lesions characterized by diffuse infiltration of the lung parenchyma by small lymphocytes, monocytoid cells and plasma cells (Fig. 2). Immunohistochemical staining demonstrated that the cells were positive for CD20 and B-cell lymphoma 2 protein, with 10% nuclear staining for Ki-67. According to the pathological findings, the patient was treated with 6 courses of chemotherapy with 2 mg vincristine, 4 mg/day chlorambucil plus 30 mg/day prednisolone for 7 days. After 2 cycles of chemotherapy, all the pulmonary lesions were significantly reduced in size (Fig. 3). Complete remission of the lung consolidations was observed following 6 cycles of treatment, as visualized by CT scan (Fig. 4). Fifteen months after the last course of chemotherapy, the patient was in good condition without any evidence of relapse. The patient remains in follow-up.

## Discussion

Although pMALToma is the most common form of primary pulmonary lymphoma, it is a rare disease. The median age at diagnosis for pMALToma is 50–60 years (5), with only few patients aged <30 years (6). pMALToma may be comorbid with autoimmune or infectious diseases (3,4). It has been reported that pMALToma is associated with Sjögren’s syndrome, rheumatoid arthritis, dysgammaglobulinemia, amyloid deposits, collagen vascular diseases, *Helicobacter pylori* infection and acquired immune deficiency syndrome (7–10). While the clinical manifestations are usually non-specific, including cough, mild dyspnea, chest pain and occasionally hemoptysis, the majority of the patients are asymptomatic, as in the presently reported case. Therefore, symptoms and physical signs contribute little to diagnosis (3). Patients with pMALTomas typically present with single or multiple radiologically detected pulmonary nodules or consolidations (8). A definitive diagnosis of pMALToma may be achieved following histological examination of biopsy specimens obtained via minimally invasive procedures, including transbronchial biopsy or radiologically-guided transthoracic core-needle biopsy. However, the tissue sample is occasionally inadequate for diagnosis, particularly in patients with atypical CT findings. Therefore, several patients are diagnosed based on the results of surgical biopsies (7,11). In addition, immunohistochemical staining findings are crucial for accurate diagnosis.

pMALTomas typically have an indolent course and a good prognosis, although systemic dissemination and transformation into high-grade B-cell lymphoma may occur. Borie *et al* (5) reported the clinical characteristics and prognostic factors of 63 cases with pMALToma; in that cohort of patients, the estimated 5- and 10-year overall survival rates were 90 and 72%, respectively. The optimal therapy for this rare disease remains under debate, mainly due to the limited availability and heterogeneity of the data reported in the literature (11–13). Various therapeutic regimens including radiotherapy, surgery and chemotherapy have been proposed. Troch *et al* (14) suggested that a watch-and-wait policy may be adopted as the initial management of pMALToma in the absence of symptoms. By contrast, Ahmed *et al* (11) reported 22 cases of biopsy-proven pMALTomas. Among those cases, 6 patients were initially observed for a median duration of 18 months (range, 10–53 months), 4 of whom ultimately received treatment due to disease progression. In addition, lung surgery may not be the optimal option, as thoracic pain and lung function impairment are observed in ~10–15% of the cases (15,16). Radiation therapy for pMALToma is also avoided, in order to prevent potential problems with lung function (16). Chemotherapy with chlorambucil currently appears to be the optimal treatment option for cases with disseminated disease (5). The therapeutic role of rituximab remains unclear, since thus far there is only a limited number of case reports with conflicting results (14,16–17). However, as pMALToma cells express the CD20 antigen, the therapeutic possibility of rituximab appears to be promising, either in combination with chemotherapy or as a single agent. Recently, Zinzani *et al* (12) reported an observational retrospective study on homogeneous clinical data from 17 patients with pMALToma; all the patients were treated with fludarabine and mitoxantrone-containing regimens. The immunochemotherapy mentioned above achieved a high response rate: 82.3% of the patients achieved a complete response and 11.8% a partial response. Furthermore, a remarkable progression-free survival (71%) and overall survival (100%) were reported at 14 years. The approach to the case presented here was treatment with chlorambucil combined with prednisolone and vincristine, which was effective in achieving a complete remission.

In conclusion, pMALToma is a rare disease and its progression is generally indolent. pMALToma may be accompanied by autoimmune diseases. We presented the case of a 19-year-old patient with biopsy-proven diagnosis of pMALToma who had no history of autoimmune disease. The patient was successfully treated with a chlorambucil-based regimen. However, long-term follow-up is required to establish the efficacy of this treatment strategy.

## Figures and Tables

**Table I:** Pertinent laboratory data at the time of the patient’s initial evaluation.

Laboratory data	Admission value	Reference range
White blood cell count/μl	5,100	4,000–10,000
Erythrocyte sedimentation rate, mm/h	23	0–20
Antinuclear antibody titer	Negative	Negative
β_2_-microglobulin, mg/l	1.44	0.91–2.2
Lactate dehydrogenase, IU/l	121	91–180
Aspartate aminotransferase, IU/l	22	0–40
Alanine aminotransferase, IU/l	23	0–40
